# Determinants of the severity of sports injuries among students in sports disciplines at higher education institutions

**DOI:** 10.3389/fpubh.2025.1565393

**Published:** 2025-06-09

**Authors:** Mingxi Li, Junwei Wang, Jisheng Xu, Yi Jia

**Affiliations:** ^1^School of Physical Education, North University of China, Taiyuan, Shanxi Province, China; ^2^School of Mathematics, Southwest Jiaotong University, Chengdu, Sichuan Province, China; ^3^School of Sports Medicine and Health, Chengdu Sport University, Chengdu, Sichuan Province, China

**Keywords:** university students, sports discipline, sports injuries, influencing factors, body mass index

## Abstract

**Introduction:**

This study aims to analyze and identify the main factors influencing the severity of sports injuries among university sports students.

**Methods:**

The study involved students aged18.5 to22.2 years from five sports programs in Sichuan Province. Data were gathered via online questionnaires and interviews in February-March2024, assessing personal characteristics and fitness indicators, with binary logistic regression applied to analyze injury severity.

**Results:**

A total of 1,190 valid responses were collected, comprising 837 males and 353 females. Univariate analysis showed that BMI, time of day, temperature perception, adherence to technique, warm-up activities, physiological condition, training duration, and vital capacity were statistically significant. Binary logistic regression analysis indicated. Factors significantly associated with increased severity of sports injuries included body mass index (BMI) categorized as underweight or overweight (OR = 1.761, *p* = 0.001), afternoon training sessions (OR = 1.499, *p* = 0.012), evening training sessions (OR = 1.888, *p* = 0.003), presence of illness or injury (OR = 1.785, *p* = 0.004), training duration of 12 h or more per week (OR = 1.355, *p* = 0.047) were positively correlated with the occurrence of severe sports injuries, and minimal warm-up activities (OR = 0.701, *p* = 0.015) was negatively correlated with the occurrence of severe sports injuries.

**Conclusion:**

BMI, training time, physiological state, weekly training duration, and warm-up practices significantly affect sports injury severity. Educational institutions should develop informed strategies in academic and training programs to reduce injury severity and ensure student health and safety.

## 1 Introduction

Sports injuries are an inevitable consequence of high-intensity athletic activities, with severe injuries posing significant risks not only to athletic performance but also to long-term health. Students in sports disciplines at higher education institutions, who undergo frequent and intense training and competition, are particularly susceptible to various types of sports injuries. Severe injuries can jeopardize students’ physical health and potentially impact their academic and professional futures, leading to substantial economic and psychological burdens for individuals and their families (1). Despite increasing attention to the health of university students, the elevated risk of sports injuries among physical education majors remains a significant concern. An epidemiological study reported that 124 first-year university physical education teacher education students sustained 107 injuries during their initial year, with 53% occurring during course-related activities (2). More recent research identified 2,326 new sports injuries among 1,332 physical education majors over the course of their university education, with injury distribution varying according to academic year, activity setting, and sport-specific demands (2). Overall, previous studies have indicated that sports-related injury rates among sports majors are higher compared to the general physically active population, underscoring the importance of implementing preventive measures (3). Investigating the characteristics and influencing factors of sports injuries, as well as developing targeted prevention and intervention strategies, is essential for effectively reducing injury incidence and promoting the long-term health and career development of physical education students.

Students in sports disciplines often face an inherent risk of sports injuries due to their frequent participation in athletic activities. The occurrence of such injuries is influenced by a complex interplay of factors. Previous research has identified several key contributors, including gender, psychological factors, physical fitness, and the sports environment (4–9). Previous research has identified several factors associated with sports injuries. Studies have indicated that female athletes may be at a higher risk of injury in certain sports (4). Psychological factors such as stress, anxiety, and depression can also impair attention and reaction times, thereby increasing the risk of injury (5). Inadequate management of training loads and lack of periodized training plans can lead to cumulative fatigue, reduce the resilience of muscles and joints, and elevate the risk of severe injuries (6). The types and incidences of injuries vary by sport; for instance, knee injuries are common among soccer players, while head injuries are more frequent among boxers (4). High-intensity and prolonged training or competition further exacerbate the risk of injuries, especially when athletes have not fully recovered (7). Unsafe or uneven playing surfaces increase the likelihood of ankle sprains and other injuries (8). Additionally, the use of inappropriate or poor-quality sports equipment can also heighten the risk of injury (9). Although existing research has examined the factors influencing sports injuries among college students majoring in sports, several limitations remain. Most previous research has focused on professional athletes, while university sports students often lack similar levels of attention and resource support. Moreover, the factors influencing the severity of athletic injuries during training and competition possess distinct characteristics, meaning that prevention strategies developed for professional athletes may not be entirely applicable or effective for sports majors. Additionally, most existing studies have remained at the descriptive or comparative level, with few employing mathematical models to elucidate the interrelationships between injury risk factors and injury outcomes.

Therefore, investigating sports injuries and their determinants specifically among university sports students not only helps to address this research gap but also provides a scientific basis for developing more effective preventive and intervention strategies by university sports departments. This study aims to comprehensively assess the severity of sports injuries among students specializing in sports disciplines by analyzing multiple influencing factors, with the goal of identifying key factors that significantly affect the severity of severe injuries.

## 2 Materials and methods

### 2.1 Data sources

Using a stratified sampling method, we recruited participants from various geographic regions across Sichuan Province, ensuring diversity in geographic distribution, economic development levels, and sports participation habits. Ultimately, the study targeted students majoring in sports disciplines from five universities in Sichuan Province, enrolled from 2019 to 2022. Surveys were distributed online to students via the WeChat Mini Program “Wenjuanxing,” strict privacy protection measures were implemented during data collection. Inclusion criteria were: Students aged between 18 and 25 years, who are in good health, without chronic diseases or other health issues that affect normal physical activity; students currently enrolled in sports programs at universities; have participated in at least one physical fitness test and one sports competition in the past year; engage in a minimum of 5 h of sports training per week during their time at school. Exclusion criteria included: Have a history of major surgery or severe injuries, especially those affecting physical function; incomplete questionnaires or those with numerous missing or duplicated responses, students who withdrew from the university or took a leave of absence due to injury or non-sport-related reasons during the study period, students who did not participate in any sports activities or training during their university years, and those who refused to provide complete physical assessment data or participate in the survey for personal reasons.

For the valid questionnaires, physical assessment scores from the university’s data platform were extracted to provide data on personal characteristics and physical fitness indicators for this study. The occurrence of sports injuries was noted as having occurred during the students’ time at university.

### 2.2 Participant characteristics

A total of 1,256 questionnaires were distributed, and after excluding those with missing or duplicated responses, 1,190 valid questionnaires were obtained, yielding a response rate of 94.74%. Descriptive analyses of the participants’ demographic and professional characteristics are presented in Tables 1, 2. The age range of participants was from 17.5 to 19.2 years. Among the participants, 837 were male, with an average height of 180.1 cm, weight of 72.2 kg, and BMI of 22.2. The 353 female participants had an average height of 168.2 cm, weight of 58 kg, and BMI of 20.5. The students were enrolled in various sports disciplines, including sports training, ethnic traditional sports, physical education, social sports, and recreational sports. The ethnic traditional Sports major focuses on cultivating high-level talents in competitive Wushu training and popular Wushu instruction, while promoting and preserving traditional Chinese sports culture. The recreational sports major aims to develop applied professionals with skills in planning, organizing, managing, and promoting recreational sports programs, such as water sports, fitness and wellness activities, and outdoor adventure programs. These majors are classified as specialized fields within the discipline of Physical Education. Students in sports training and ethnic traditional sports had weekly training durations exceeding 12 h, followed by physical education students (7.5–12 h), with social sports and recreational sports students training 5–7.5 h per week.

**Table 1:** Demographic information.

Variable	Category	n	%
Gender	Male	837	70.3
	Female	353	29.7
Grade	First-year	704	59.2
	Second-year	169	14.2
	Third-year	153	12.9
	Fourth-year	64	5.4
Major	Sports Training	384	23.9
	Ethnic Traditional Sports	110	9.2
	Physical Education	216	18.2
	Social Sports	151	12.7
	Recreational Sports	329	27.6
Severity of Sports Injuries	Minor Injuries	885	74.4
	Severe Injuries	305	25.6

**Table 2:** Participant characteristics.

Variables	Male (*n* = 837)	Female (*n* = 353)
Height (cm)	177.9 + 6.8	165.5 + 6.4
Weight (kg)	69.4 + 8.5	56.4 + 7.8
BMI	21.9 ± 2.1	20.6 ± 2.5

### 2.3 Questionnaire development

Based on the academic literature on sports injuries among university students and young athletes, consultations with experts in the field, combined with online resources, personal experiences, and interviews with peers, a questionnaire was meticulously compiled to explore the factors related to sports that influence sports injuries. The study content covers common types of sports injuries and their influencing factors, ensuring scientific rigor and applicability. The independent variables were categorized into three main types: 1. Personal Characteristics and Physical Fitness Indicators: Body Mass Index (BMI), vital capacity, and sit-and-reach flexibility rating, among others. 2. Sports Environment and Behavior: Injury scenarios, surface material, weather conditions, and season. 3. Psychological and Physiological State: Warm-up activities, perceived stress, and physiological condition.

The International Severity Score (ISS) was used to assess the severity of injuries reported by participants. Each student’s injury score was calculated based on the ISS. Injuries were categorized as follows: minor injuries were defined as ISS scores ≤16; severe injuries were defined as ISS scores >16 and ≤25; and critical injuries were defined as ISS scores >25. Due to the low incidence of critical injuries in the sample, severe and critical injuries were combined for analytical simplicity and statistical validity, resulting in a final classification of injuries as either minor (ISS ≤ 16) or severe (ISS > 16).

To ensure the quality and reliability of the questionnaire and data, multiple expert reviews and revisions were conducted during the initial design phase of the questionnaire. Before the official distribution, a small-scale pilot survey was carried out, and the questionnaire content was further optimized based on the analysis of the pilot results. During the data collection process, all questionnaires were completed under the supervision of teachers to ensure that students responded truthfully and independently.

### 2.4 Statistical methods

Descriptive statistics were used to summarize categorical variables. Univariate analyses were conducted using chi-square tests to assess the risk factors potentially influencing the severity of sports injuries. Variables showing statistical significance at the chi-square test level (*p* < 0.05) were further analyzed using a binary logistic regression model to evaluate the impact of these factors on injury severity. Odds ratios (OR) and 95% confidence intervals (CI) were calculated as estimates of effect size. Statistical significance was considered at *p* < 0.05.

## 3 Results

### 3.1 Univariate analysis of factors

A univariate analysis of 19 influencing factors on the severity of sports injuries identified 8 categorical variables with statistical significance (*p* < 0.05): BMI and vital capacity under personal characteristics and physical fitness indicators; time of day, temperature perception, and adherence to proper technique under sports environment and behavior; and warm-up activities, physiological condition, and training duration under psychological and physiological state. A statistically significant difference in the incidence of sports injuries was found across different training times of day (χ^2^ = 12.88, *p* = 0.002), with the highest rate occurring during the afternoon. Significant differences were also observed in injury incidence among physical education majors based on the adequacy of warm-up activities (χ^2^ = 13.50, *p* = 0.001); students who performed inadequate warm-ups were more likely to sustain injuries. Similarly, training duration was significantly associated with injury incidence (χ^2^ = 12.76, *p* = 0.002), with the highest rate observed among those training more than 12 h per week. These findings are detailed in Table 3.

**Table 3:** Comparison of injury severity by category.

Category	Subcategory	Severity of sports injuries		Chi-square value	*p*-value
		Minor (*n* = 885)	Severe (*n* = 305)		
Injury Scenario	In-Class and Extracurricular Activities	331(37.4)	120(39.3)	1.762	0.414
	Professional Training	414(46.8)	130(42.6)		
	Sports Competitions	140(15.8)	55(18)		
Venue Standard	Standard Sports Venue	661(74.7)	217(71.1)	1.471	0.228
	Non-Standard Sports Venue	224(25.3)	88(28.9)		
Weather	Sunny	520(58.8)	200(65.6)	4.952	0.084
	Cloudy	261(29.5)	71(23.3)		
	Rainy	104(11.8)	34(11.1)		
Season	Spring	157(17.7)	43(14.1)	2.943	0.401
	Summer	268(30.3)	94(30.8)		
	Autumn	140(15.8)	57(18.7)		
	Winter	320(36.2)	111(36.4)		
Time of Day	Morning	322(36.4)	78(25.6)	12.881	0.002**
	Afternoon	449(50.7)	174(57)		
	Evening	114(12.9)	53(17.4)		
Temperature Perception	Moderate	417(47.1)	155(50.8)	9.054	0.011*
	Cold	344(38.9)	92(30.2)		
	Hot	124(14)	58(19)		
Type of Sport	Non-Contact Sports	308(34.8)	112(36.7)	1.081	0.583
	Indirect Contact Sports	462(52.2)	149(48.9)		
	Direct Contact Sports	115(13)	44(14.4)		
Adherence to Technique	Proper	516(58.3)	204(66.9)	6.295	0.043*
	Improper	369(41.7)	101(33.1)		
Interference	None	485(54.8)	148(48.5)	3.59	0.058
	Present	400(45.2)	157(51.5)		
Warm-Up Activities	Sufficient	316(35.7)	145(47.5)	13.502	0.001**
	Insufficient	466(52.7)	133(43.6)		
	None	103(11.6)	27(8.9)		
Perceived Stress	Yes	106(12)	38(12.5)	0.049	0.824
	No	779(88)	267(87.5)		
Perceived Depression	Yes	151(17.1)	45(14.8)	0.878	0.349
	No	734(82.9)	260(85.2)		
Perceived Anxiety	Yes	223(25.2)	69(22.6)	0.812	0.367
	No	662(74.8)	236(77.4)		
Physiological Condition	Healthy	595(67.2)	193(63.3)	12.072	0.002**
	Sub-Healthy	206(23.3)	61(20)		
	Injured or Ill	84(9.5)	51(16.7)		
Injury Location	Upper Limb	81(9.2)	27(8.9)	5.994	0.2
	Trunk	175(19.8)	66(21.6)		
	Lower Limb	611(69)	208(68.2)		
	Head	18(2)	4(1.3)		
Training Duration	<7.5 h/week	369(41.7)	111(36.4)	12.761	0.002**
	7.5–12 h/week	174(19.7)	42(13.8)		
	>12 h/week	342(38.6)	152(49.8)		
Vital Capacity	Excellent	103(11.6)	22(7.2)	4.725	0.03*
	Average	782(88.4)	283(92.8)		
Sit-and-Reach Flexibility	Excellent	252(28.5)	75(24.6)	1.717	0.19
	Average	633(71.5)	230(75.4)		
BMI	Normal	766(86.6)	238(78)	12.489	0.000**
	Underweight or Overweight	119(13.4)	67(22)		

*p < 0.05; **p < 0.01.

### 3.2 Binary logistic regression analysis

Using the severity of sports injuries as the dependent variable, binary logistic regression analysis was performed with the eight significant factors identified in the chi-square test, along with BMI, as independent variables (Table 4).

**Table 4:** Binary logistic regression analysis results for injury severity.

Predictor	B	S. E,	Wals	*p*	OR	95%CI
BMI						
Underweight or Overweight	0.566	0.175	10.409	0.001**	1.761	1.249 ~ 2.483
Time of Day			8.51	0.006**		
Morning					1	
Afternoon	0.405	0.161	6.333	0.012*	1.499	1.094 ~ 2.055
Evening	0.636	0.215	8.736	0.003**	1.888	1.239 ~ 2.878
Warm-Up Activities			6	0.035*		
Sufficient					1	
None	−0.414	0.248	2.772	0.096	0.661	0.406 ~ 1.076
Insufficient	−0.355	0.147	5.883	0.015*	0.701	0.526 ~ 0.934
Temperature Perception						
Moderate			5.573	0.058	1	
Cold	−0.266	0.156	2.892	0.089	0.766	0.564 ~ 1.041
Hot	0.199	0.192	1.074	0.300	1.22	0.837 ~ 1.778
Physiological Condition			10.591	0.014*		
Healthy					1	
Sub-Healthy	0.014	0.176	0.006	0.938	1.014	0.718 ~ 1.432
Illness	0.579	0.202	8.215	0.004**	1.785	1.201 ~ 2.653
Adherence to Technique						
Improper	−0.168	0.149	1.275	0.259	0.845	0.631 ~ 1.132
Vital Capacity						
Average	−0.134	0.14	0.919	0.338	0.875	0.665 ~ 1.150
Training Duration			9.198	0.019*		
<7.5 h/week					1	
7.5–12 h/week	−0.214	0.209	1.049	0.306	0.807	0.536 ~ 1.216
>12 h/week	0.304	0.153	3.931	0.047*	1.355	1.003 ~ 1.83

*p < 0.05; **p < 0.01.

Logistic regression analysis identified five statistically significant factors affecting the likelihood of severe injury. BMI demonstrated an OR of 1.761 (*p* = 0.001; 95% CI: 1.249–2.483), indicating a 76.1% increase in the probability of severe injury for individuals with either underweight or overweight compared to those with a normal weight. The risk of severe injury was significantly higher in the afternoon (OR = 1.499; 95% CI: 1.094–2.055; *p* = 0.012), with a 49.9% increased likelihood. The evening period showed an even greater risk (OR = 1.888; 95% CI: 1.239–2.878; *p* = 0.003), with an 88.8% increase in the probability of severe injury. The presence of illness or injury status was associated with an OR of 1.785 (95% CI: 1.201–2.653; *p* = 0.004), corresponding to a 78.5% increase in the risk of severe injury. A training duration of 12 h or more per week was linked to a 35.5% increased likelihood of severe injury (OR = 1.355; 95% CI: 1.003–1.830; *p* = 0.047). Compared to sufficient warm-up activities, performing only insufficient warm-up activities was associated with a 29.9% reduced probability of severe injury (OR = 0.701; 95% CI: 0.526–0.934; *p* = 0.015).

## 4 Discussion

Sports injuries are a common and significant issue among university athletes. Understanding the influencing factors of sports injuries is crucial for developing effective prevention strategies. This study explores the impact of various factors on the severity of sports injuries in physical activities, including personal characteristics and physical fitness indicators, sports environment and behavior, and psychological and physiological state. The findings of this study underscore the significant impact of BMI, activity time periods, physiological state, weekly training duration, and warm-up practices on the severity of injuries sustained during athletic training. Identifying and managing these key variables, along with implementing effective interventions, may mitigate the severe consequences of sports injuries among collegiate athletes.

In this study, BMI was notably associated with severe injuries, with both underweight and overweight conditions correlating with an increased risk of severe injury. This finding aligns with the conclusions of C. Toomey et al., who observed that high BMI is linked to an increased risk of sport-related injuries in adolescents under the age of 20, particularly lower limb injuries, with a combined OR of 1.18 (10). Another study found that among female athletes, those who sustained injuries had a significantly higher average BMI compared to their uninjured counterparts (21.9 vs. 19.5), demonstrating a marked association between higher BMI and sports injuries (11). Arbabi et al. reported that an increase in BMI is associated with elevated mortality risk and severity of lower limb injuries, indicating a complex relationship between weight and injury outcomes. The concept of a “buffering effect” suggests that overweight individuals might experience lower injury severity in certain body areas, highlighting the multifaceted impact of BMI on injury patterns (12). Regarding the impact of underweight on sports injuries, a study of Premier League professional soccer players found that athletes with low BMI are more prone to sports injuries (13). Research on male endurance athletes has shown that low BMI correlates with reduced energy availability, impacting performance and testosterone levels, thereby increasing the risk of injury and illness (14). Additionally, studies on tennis players have indicated a significant association between BMI and the risk of sports injuries, emphasizing the importance of maintaining an optimal BMI to avoid injuries related to both high and low body weight (15). In conclusion, both underweight and overweight conditions correlating with an increased risk of severe injury, suggesting that there may be a nonlinear relationship between BMI and sports injuries. This phenomenon requires further research to be validated. Collegiate athletes should aim to maintain a normal BMI to reduce the risk of injuries and decrease the likelihood of experiencing severe sports-related injuries.

In this study, we observed a 78.5% increase in the likelihood of severe injury under conditions of illness or pre-existing injury compared to a healthy state. Interviews with collegiate athletes revealed that their illness or injury conditions typically include muscle strains and sprains, joint pain, cramps and spasms, headaches and dizziness, and gastrointestinal issues. These conditions often lead to decreased athletic performance and an elevated risk of severe injury. Research by Zhou et al. suggests that engaging in sports or physical training while injured or ill may result in persistent pain and muscle atrophy (16). The pressure to continue participating in physical activities under such conditions exacerbates the decline in physical status, thereby increasing the risk of severe injury (17). A study on elite adolescent athletes found that those with prior or current injuries had a significantly higher probability of sustaining severe injuries during the season (OR = 53.9; 95% CI: 7.1–407.7) (18). Research focusing on collegiate athletes indicates that those with a history of health issues or injuries face a higher risk of severe injuries during competitions, particularly non-contact injuries such as ankle sprains and anterior cruciate ligament tears (19). Moreover, psychological factors such as stress, anxiety, and depressive symptoms significantly exacerbate under illness or injury conditions, further affecting athletes’ recovery and risk of re-injury (20). Evidence indicates that health issues or pre-existing injuries substantially increase the risk of severe injuries among young elite athletes, which not only impacts athletic performance but also potentially leads to future disability and reduced activity levels (21). In conclusion, health problems or injury status significantly increase the risk of severe sports injuries among collegiate athletes. Effective management and intervention targeting these risk factors are crucial to safeguarding athlete health and performance.

In this study, the likelihood of severe injury was 49.9% higher in the afternoon and 88.8% higher in the evening compared to morning activities. Research by Chien-Chun Chang highlights the significant impact of strenuous training and fatigue accumulation on the risk of injury (22). Interviews with students and coaches revealed that students usually have intensive academic tasks in the morning, which can lead to accumulated fatigue. High-intensity training is often scheduled for the afternoon and evening. The accumulated fatigue from the morning, combined with the additional stress from high-intensity training, can significantly increase the risk of severe sports injuries in the afternoon and evening. Diminished lighting at night can impair visual judgment, while lower temperatures may lead to muscle stiffness (23). Furthermore, the body’s circadian rhythm may influence athletic performance and injury risk (24–26). Typically, body temperature is higher later in the afternoon, which may enhance physical function but could also result in overconfidence or increased fatigue. These factors may collectively elevate the risk of severe injuries. Research by Simo Salminen has identified evening activity, prolonged working hours, shift work, and inadequate warm-up as risk factors for occupational and sports-related injuries (27). These findings suggest that conditions associated with evening sports activities may lead to a heightened risk of injury.

Compared to students with weekly training durations of less than 7.5 h, those with more than 12 h of training per week experience a 41% increased likelihood of severe injury. Collegiate athletes face dual pressures from academic and training demands, often leading to irregular schedules with late nights and inconsistent sleep patterns. This situation may result in insufficient recovery for those with higher training volumes, particularly during consecutive training days. Without proper load management and periodized training plans, accumulated fatigue can reduce the stress tolerance of muscles and joints, thereby elevating the risk of severe injuries. Research on elite rugby players indicates that improper management of training volume and lack of periodized training contribute to cumulative fatigue, increasing the risk of injuries (28). Inadequate training load management and lack of periodization can lead to overtraining syndrome, characterized by chronic fatigue and an elevated risk of injuries due to accumulated fatigue (29, 30). Another study highlights a potential positive correlation between training duration and injury severity among collegiate athletes, noting that increased training time is associated with a higher severity of sports injuries (31). Additionally, a cross-sectional study of collegiate athletes found a significantly higher incidence of sports injuries during college compared to high school, emphasizing that increased training duration may lead to a greater risk of injuries (32). Research on young soccer players suggests that increased training duration may be related to a higher risk of severe sports injuries, particularly under certain risk factors and specific characteristics (33). Moreover, students with longer training times may experience greater psychological stress and anxiety, which can impair concentration and judgment, indirectly raising the risk of severe injuries during sports (34, 35). In summary, among collegiate athletes facing dual pressures from academics and training, increased training duration may significantly elevate the risk of severe injuries. This risk is related not only to accumulated fatigue and inadequate recovery but also to irregular schedules and poor dietary habits. Strengthening training load management, implementing scientific periodized training plans, and addressing dietary and psychological health are crucial measures to reduce the risk of sports injuries.

Warm-up is defined as low- to moderate-intensity physical activity performed several minutes to a few hours before exercise, intended to enhance performance and reduce the risk of sports injuries, thereby facilitating optimal athletic outcomes (36). Existing studies have identified several mechanisms through which warm-up benefits the human body, including elevated body temperature, increased metabolic activity, and enhanced neurological and psychological readiness, all of which contribute to improved performance and a lower incidence of injuries (36, 37). However, in our study, adequate warm-up did not reduce the incidence of sports injuries among physical education majors, a finding contrary to conventional expectations. This result may be attributed to various factors. Warm-up consists of multiple components—such as the type of exercise, intensity, and duration—and its effectiveness is influenced by additional variables, including the sport type, individual athlete characteristics, and competitive environment (38). Specifically, for short-duration activities (≤10 s), a 10–20 min warm-up at 60% of maximal oxygen uptake may maximize body temperature and minimize phosphagen depletion. In contrast, for prolonged activities (>300 s), warm-ups exceeding 10 min may reduce muscle energy reserves and heat tolerance (39). In conclusion, both insufficient preparation and overly intense warm-up may compromise its intended benefits. Furthermore, one possible explanation is that the sports activities undertaken following insufficient warm-up might be inherently lower in risk. Additionally, insufficient warm-up may lead to heightened psychological tension and concentration among athletes, whereas extended warm-up sessions might induce psychological fatigue or reduced alertness, thereby potentially affecting performance and safety (40). In situations involving insufficient warm-up, coaches might place greater emphasis on supervision and immediate feedback to ensure athletes achieve optimal performance within the limited preparation time. Moreover, insufficient warm-up may lead athletes to rely more on technical execution rather than physical conditioning, particularly in sports that are highly technical (41). Conversely, sufficient warm-up might cause some athletes to become overly confident in their technical execution, potentially overlooking crucial technical details and increasing the risk of injury.

This study acknowledges several limitations. First, the subjective nature of survey responses may introduce bias, as students might be influenced by social expectations or personal psychological states, potentially affecting the accuracy and objectivity of their self-reported data. Additionally, the reliance on participants’ memory for reporting sports injuries could result in recall bias. Future research should consider incorporating objective measurement indicators to minimize recall bias. Second, while the study explored various factors potentially influencing sports injuries, the investigation may not be comprehensive. For instance, the survey did not utilize more precise psychological scales to assess psychological states prior to injury, which may limit the thoroughness and accuracy in evaluating the role of psychological factors. This oversight could result in missing significant potential influences. Furthermore, the study lacked a comprehensive evaluation of factors such as athletes’ competitive level, training environment, and equipment safety, which are critical for understanding the complexity of sports injuries. In addition, although stratified sampling was used to enhance sample diversity, voluntary participation may have introduced self-selection bias—for example, individuals with greater concern for sports health were more likely to complete the questionnaire. Furthermore, the sample size and regional distribution in this study were limited, which may constrain the generalizability and external validity of the findings. This study identifies warm-up activities as a significant factor influencing the severity of sports injuries. However, it is important to note that athletes engaging in minimal warm-up may be involved in lower-risk activities, which could partially account for the reduced incidence of severe injuries. Thus, the effect of warm-up activities may be moderated by the type of activity performed. The study did not adequately control for activity type as a confounding variable, which may affect the accurate assessment of the independent impact of warm-up activities. Future research should explore the interaction effects between warm-up activities and activity types to gain a more comprehensive understanding of their influence on sports injuries. Some classifications in this study were based on existing standards and adapted to the characteristics of the study population. For example, BMI was categorized into normal and abnormal (underweight or overweight), injury severity was classified into severe and mild based on a scale, and measures such as vital capacity and sit-and-reach flexibility were categorized as excellent or average according to national standards. While these classifications reflect the specific context of collegiate athletes, they may reduce comparability with other studies. Additionally, the choice of standards and classifications may influence the interpretation and generalization of the results. Furthermore, the data analysis methods used in the study have limitations. The cross-sectional design of the study makes it challenging to establish causality. Future research should consider prospective or longitudinal study designs to more accurately determine the causal relationships between factors and sports injuries.

This study reveals that BMI, activity timing, physiological condition, weekly training duration, and warm-up practices significantly influence the severity of sports injuries during training. It is recommended that collegiate sports departments consider individual physical conditions when designing training programs, appropriately schedule training times, monitor athletes’ physiological states, adjust training durations, and optimize warm-up activities. Future research should further investigate the specific mechanisms underlying these factors and develop targeted interventions to effectively reduce the severity of sports injuries, thereby enhancing overall training effectiveness and safety for athletes.

## 5 Conclusion

This study identifies several key factors that significantly influence the severity of sports injuries during training, including BMI, training time, physiological condition, weekly training volume, and warm-up practices. It is recommended that collegiate sports departments tailor training programs to individual physical profiles and relevant injury-related factors, appropriately schedule training sessions, monitor athletes’ physiological states, adjust weekly training loads, and optimize warm-up protocols. Although this study examined a range of factors affecting sports injuries, the reliance on self-reported data and the limited geographic scope may have influenced the findings. Future research should consider prospective or longitudinal study designs to investigate the causal relationships and underlying mechanisms of these factors, and to develop targeted intervention strategies that effectively reduce injury severity and improve training safety and efficacy.

## Data Availability

The original contributions presented in the study are included in the article/supplementary material, further inquiries can be directed to the corresponding author.
